# HR-MAS MR Spectroscopy of Breast Cancer Tissue Obtained with Core Needle Biopsy: Correlation with Prognostic Factors

**DOI:** 10.1371/journal.pone.0051712

**Published:** 2012-12-14

**Authors:** Ji Soo Choi, Hyeon-Man Baek, Suhkmann Kim, Min Jung Kim, Ji Hyun Youk, Hee Jung Moon, Eun-Kyung Kim, Kyung Hwa Han, Dong-hyun Kim, Seung Il Kim, Ja Seung Koo

**Affiliations:** 1 Department of Radiology, Research Institute of Radiological Science, Yonsei University College of Medicine, Seodaemun-gu, Seoul, Korea; 2 Department of Radiology, National Cancer Center, Ilsandong-gu, Goyang-si Gyeonggi-do, Korea; 3 Division of Magnetic Resonance, Korea Basic Science Institute, Yuseong-gu, Daejeon, Korea; 4 Department of Chemistry and Chemistry Institute for Functional Materials, Pusan National University, Geumjeong-gu, Busan, Korea; 5 Department of Research Affair, Yonsei University College of Medicine, Seodaemun-gu, Seoul, Korea; 6 College of Electrical & Electronic Engineering, Yonsei University, Seodaemun-gu, Seoul, Korea; 7 Department of Surgery, Yonsei University College of Medicine, Seodaemun-gu, Seoul, Korea; 8 Department of Pathology, Yonsei University College of Medicine, Seodaemun-gu, Seoul, Korea; Dartmouth, United States of America

## Abstract

The purpose of this study was to examine the correlation between high-resolution magic angle spinning (HR-MAS) magnetic resonance (MR) spectroscopy using core needle biopsy (CNB) specimens and histologic prognostic factors currently used in breast cancer patients. After institutional review board approval and informed consent were obtained for this study, CNB specimens were collected from 36 malignant lesions in 34 patients. Concentrations and metabolic ratios of various choline metabolites were estimated by HR-MAS MR spectroscopy using CNB specimens. HR-MAS spectroscopic values were compared according to histopathologic variables [tumor size, lymph node metastasis, histologic grade, status of estrogens receptor (ER), progesterone receptor (PR), HER2 (a receptor for human epidermal growth factor), and Ki-67, and triple negativity]. Multivariate analysis was performed with Orthogonal Projections to Latent Structure-Discriminant Analysis (OPLS-DA). HR-MAS MR spectroscopy quantified and discriminated choline metabolites in all CNB specimens of the 36 breast cancers. Several metabolite markers [free choline (Cho), phosphocholine (PC), creatine (Cr), taurine, myo-inositol, scyllo-inositol, total choline (tCho), glycine, Cho/Cr, tCho/Cr, PC/Cr] on HR-MAS MR spectroscopy were found to correlate with histologic prognostic factors [ER, PR, HER2, histologic grade, triple negativity, Ki-67, poor prognosis]. OPLS-DA multivariate models were generally able to discriminate the status of histologic prognostic factors (ER, PR, HER2, Ki-67) and prognosis groups. Our study suggests that HR-MAS MR spectroscopy using CNB specimens can predict tumor aggressiveness prior to surgery in breast cancer patients. In addition, it may be helpful in the detection of reliable markers for breast cancer characterization.

## Introduction

Breast cancer is one of the most common cancers and is an important cause of cancer related deaths among women globally [1], [2]. Early diagnosis of breast cancer is crucial for successful treatment and screening programs using mammography, ultrasonography, and magnetic resonance imaging (MRI) have been effective in industrialized countries [3]–[6]. In addition to early detection, clinical and histologic assessments of breast cancer are also important components in treatment and management. The presence of hormone receptors is a generally favorable sign of prognosis, as appropriate hormone therapy can suppress the growth of such tumors. Patients with large tumors and axillary lymph node metastasis are considered to be at high risk of tumor recurrence, and these patients require more extensive treatment. Therefore, the identification of reliable markers to improve diagnostic accuracy and predict prognosis would be an important accomplishment.

High-resolution magic angle spinning (HR-MAS) magnetic resonance (MR) spectroscopy has been applied in studies of various human tissues and diseases [7]–[10]. The HR-MAS MR spectra of tissue samples consist of multiple peaks that provide information on their metabolic composition. The assessment of tumor metabolic composition with HR-MAS MR spectroscopy has been suggested as a promising tool in the diagnosis and characterization of breast cancer [11]–[13]. Recent studies using HR-MAS MR spectroscopy have also reported that the choline-containing compounds of breast cancer tissue, including glycerophosphocholine (GPC), phosphocholine (PC), free choline (Cho), and taurine (Tau), have different concentrations and distributions according to clinicopathologic parameters associated with tumor aggressiveness [14]–[16]. However, these studies performed HR-MAS MR spectroscopy using surgically obtained tissue specimens. Therefore, their results could not be directly applied to the preoperative decision making stage at which treatment approaches are planned for patients with breast cancer.

Percutaneous image-guided core needle biopsy (CNB) is a minimally invasive and widely used procedure for the diagnosis of breast cancer prior to surgery [17]. Tissue samples obtained with CNB are clinically useful not only for pathologic diagnosis but also for immunohistochemical (IHC) analysis of histologic prognostic factors such as hormone receptor status [18]. Recently, Li et al. performed HR-MAS MR spectroscopy on breast tissue samples obtained with CNB, and showed that this approach has the potential to discriminate between cancer and non-cancer samples [19].

Therefore, we hypothesized that HR-MAS MR spectroscopy performed on breast cancer tissues obtained via CNB may correlate with histologic prognostic factors and that it may help identify reliable markers for the characterization of breast cancer. The purpose of this study was to determine whether there are any correlations between HR-MAS MR spectroscopy data for CNB specimens and histologic prognostic factors currently used in breast cancer patients.

## Materials and Methods

### Patients

This study was approved by the institutional review board of Yonsei University College of Medicine, and written informed consent was obtained from each patient prior to study commencement. Between August 2010 and February 2011, 38 female patients (mean age 52.2 years; age range 34–68 years) with a total of 40 breast lesions as assessed by the Breast Imaging Reporting and Data System (BI-RADS) staged 4c or 5 and larger than 1 cm in diameter on mammographic or US imaging were initially enrolled. All patients underwent ultrasound (US)-guided CNB for diagnosis. Of these, 34 patients with 36 breast lesions fulfilled the following inclusion criteria: 20 years of age or older; having a breast lesion pathologically diagnosed as malignant by core biopsy; not pregnant at the time of diagnosis; no history of breast cancer or previous breast surgery including breast implants. We performed HR-MAS MR spectroscopy on the CNB specimens from the 36 malignant breast lesions.

### ^1^H NMR Spectroscopy using Biopsy Specimens

The US-guided CNBs were performed with a 14-gauge dual-action semiautomatic core biopsy needle (Stericut with coaxial guide; TSK Laboratory, Tochigi, Japan) by one of four radiologists (with 6–13 years of experience). In large cancers with a heterogeneous nature, the homogeneously solid areas were targeted for biopsies. The mean number of tissue samples obtained by US-guided CNB was six (range 5–8) core samples. For HR-MAS MR spectroscopy, one core tissue sample was put in a cryogenic vial and immersed in liquid nitrogen immediately after biopsy. Samples were stored at -162°C for one to five months prior to HR-MAS MR spectroscopy.

**Figure 1:**
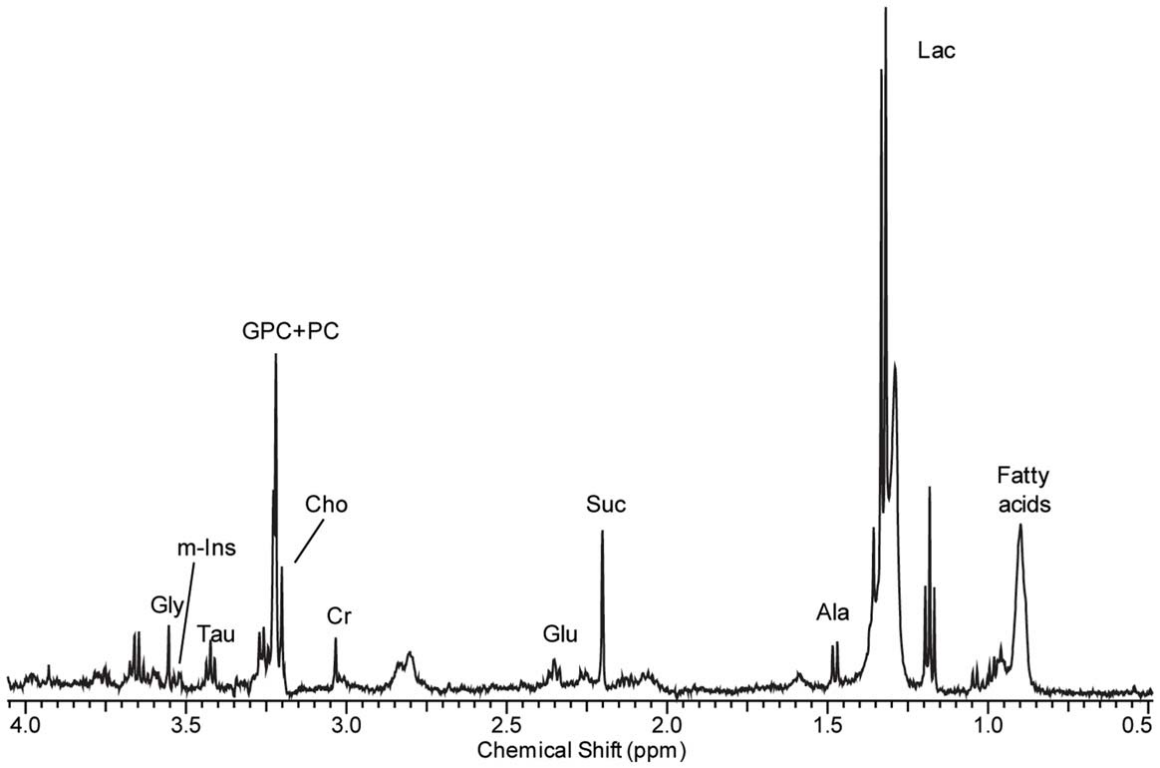
A 38-year-old woman with invasive ductal carcinoma (tumor size 37 mm, triple negative, strongly positive Ki-67). The HR-MAS MR spectrum (11.7T) obtained using the core needle biopsy specimen shows peaks of each choline metabolite. The tCho concentration measured with HR-MAS MR spectroscopy was 6.5 mmol/kg. Note. Lac, lactate; Ala, alanine; Glu, glutamate; Cr, creatine; Cho, free choline; GPC, glycerophosphocholine; PC, phosphocholine; tCho, total choline, sum of Cho, PC, and GPC; Tau, taurine; m-Ins, myo-inositol; Gly, glycine.

HR-MAS MR spectroscopy was performed on the tissue samples with an NMR (nuclear magnetic resonance) spectrometer (Agilent, VNMRS 500) operating at a proton NMR frequency of 500.13 MHz (11.74T). The temperature was set to 19°C after calibration with methanol. The examination time of each sample was approximately 1 hour. Frozen samples were thawed in the NMR laboratory, weighed, and placed in a HR-MAS nano-probe® (Agilent, Walnut Creek, CA, USA). The total volume of the sample cell was 40 ml, and an average of 7.5 mg core-biopsy samples were put in the cell with the remaining volume filled with D_2_O containing 0.01% trimethylsilyl propionic acid (TSP). An inverse-detection type probe was equipped with a single Z-gradient coil was used. The spectra were acquired with a CPMG pulse sequence to impose a T2 filter. The total T2 delay was set to 290 msec and the samples were spun at 2 KHz. The spectra were acquired with total complex points of 16 K, sweep width of 7961 Hz, and 1024 transients. The 90 degree pulse was calibrated with each sample on water resonance. The water signal was saturated using a continuous weak power wave during the recycle delay. Each free induction decay signal was processed and analyzed using ACD software (Advanced Chemistry Development, Toronto, Ontario, Canada). Post-processing consisted of Fourier transformation, phasing and baseline correction. Chemical shifts were referenced in relation to the creatine (Cr) signal at 3.04 ppm. Spectral regions from 1.47 to 3.60 ppm [alanine (Ala), succinate (Suc), Cr, Cho, PC, GPC, myo-inositol (m-Ins), scyllo-inositol (s-Ins), Tau, glycine (Gly)] were selected for quantification (Fig. 1). Metabolite peak amplitudes were measured by fitting a Voigt (e.g., Gauss+Lorentz) lineshape function. The integration values were normalized to the number of contributing protons per molecule and to tissue weight. Quantification was performed by comparing the integrated TSP signal with the signal of interest in the tumor spectrum. Absolute concentrations were recorded as means±SD in µmol/g wet weight.

### Histopathologic Analysis

All 36 lesions were pathologically diagnosed as malignant by CNB prior to treatment. The final diagnosis was established with surgery in 29 patients with 31 malignant lesions (one patient had bilateral breast cancers and one patient had multicentric breast cancers found in two quadrants of the breast). In the remaining five patients with five malignant lesions, the final diagnosis was confirmed by CNB without surgery. The final histopathologic results were used as reference standards. Information about pathologic variables including histologic grade, estrogen receptor (ER), progesterone receptor (PR), HER2 (a receptor for human epidermal growth factor), and Ki-67 status was based on final pathologic reports.

All tissues were fixed in 10% buffered formalin and embedded in paraffin. All archival hematoxylin and eosin (H&E)-stained slides for each case were reviewed by experienced pathologists. The histologic grade of each tumor was determined with the modified Bloom-Richardson classification [20].

IHC analyses for ER, PR, HER2, and Ki-67 were performed on tissue blocks. Briefly, 5 µm-thick sections were obtained with a microtome, deparaffinized and rehydrated. After a 10 minute treatment with 3% hydrogen peroxide solution to block endogenous peroxidases, the sections were pretreated in 10 mM citrate buffer (pH 6.0) for antigen retrieval in a microwave oven for 20 minutes. After incubation with primary antibodies against ER (clone SP1, 1∶100; Thermo Scientific, Fremont, CA, USA), PR (clone PgR 636, 1∶50; DAKO, Glostrup, Denmark), HER2 (polyclonal, 1∶1500; DAKO), and Ki-67 (1∶400 dilution; Novocastra, Newcastle, U.K.), immunodetection was performed with biotinylated antimouse immunoglobulin, followed by peroxidase-labeled streptavidin using a labeled streptavidin biotin kit with 3,3′-diaminobenzidine chromogen as the substrate. Slides were counterstained with Harris hematoxylin. ER and PR positivity was defined as more than 10 fmol/mg cytosol protein, or as 10% or more nuclear IHC staining. IHC stain results of HER2 were scored by counting the number of cells positively stained on the membrane and expressed as a percentage of total tumor cells. HER2 staining was scored as follows: 0, membrane staining in <10% of tumor cells; 1+, faint or incomplete membrane staining in >10% of cells; 2+, weak or moderate complete or incomplete staining in >10% of cells; 3+, strong complete membrane staining in >10% of cells. Tumors scored as 3+ were considered HER2 positive cases whereas tumors with 0 to 1+ were regarded as negative cases. Borderline cases (2+) required further investigation using fluorescence *in situ* hybridization to assess gene amplification. Triple negativity was defined as the complete lack of expression of ER, PR, and HER2. IHC staining of Ki-67 was scored by counting the number of cells with positively stained nuclei and expressed as a percentage of the total tumor cells. Staining results for Ki-67 were classified as follows: Group 1: ≤10%, Group 2∶10–29%, and Group 3: ≥30% [21].

### Data and Statistical Analysis

Patients’ clinicopathologic data was collected from a review of their medical records. Clinicopathologic variables included the pathologic type of each tumor, lymph node metastasis at the time of diagnosis, tumor size, histologic grade, status of ER, PR, HER2 and Ki-67 expression, and triple negativity (Table 1). Tumor size was based on final pathologic results (n = 22). However, tumor size measured with US was used when patients received neoadjuvant chemotherapy before surgery (n = 9) or did not undergo surgery (n = 5).

**Table 1:** Clinicopathologic data of 34 patients with 36 malignant breast lesions in this study.

Clinicopathologic variables		Patients (%)	Lesions (%)
Histologic grade	Low (Grade 1–2 )	24 (70.6)	26 (72.3)
	High (Grade 3)	10 (39.4)	10 (27.7)
Tumor size	≤2 cm	15 (44.1)	17 (47.2)
	>2 cm	19 (55.9)	19 (52.8)
ER status	Negative	6 (17.7)	6 (16.6)
	Positive	26 (76.4)	28 (77.8)
	N/A	2 (5.9)	2 (5.6)
PR status	Negative	18 (52.9)	18 (50.0)
	Positive	14 (41.2)	16 (44.4)
	N/A	2 (5.9)	2 (5.6)
HER2 status	Negative	27 (79.4)	29 (80.5)
	Positive	5 (14.7)	5 (13.9)
	N/A	2 (5.9)	2 (5.6)
Triple negativity	Yes	4 (11.7)	4 (11.1)
	No	28 (82.4)	30 (83.3)
	N/A	2 (5.9)	2 (5.6)
Ki-67 status	Low (Group 1–2)	24 (70.6)	26 (72.3)
	High (Group 3)	6 (17.7)	6 (16.6)
	N/A	4 (11.7)	4 (11.1)
Lymph node metastasis	Negative	22 (64.7)	24 (66.7)
	Positive	12 (35.3)	12 (33.3)
Prognosis	Good	7 (20.6)	9 (25.0)
	Poor	25 (73.5)	25 (69.4)

N/A: not available.

HR-MAS MR spectroscopic values (Metabolite Concentrations: Cho, PC, GPC, total choline (tCho, the sum of Cho, PC, and GPC), Cr, Gly, Tau, m-Ins, s-Ins/Metabolic Ratios: Cho/Cr, PC/Cr, GPC/Cr, GPC/PC, GPC/Cho, PC/Cho) were compared according to histopathologic prognostic factors using the Student’s *t*-test. Tumors were grouped by size based on diameter ≤2 cm or >2 cm. In addition, tumors were grouped by prognosis. The good prognosis group was defined by no spread to axillary lymph nodes, a tumor size with a diameter ≤2 cm, and positive results for ER and PR. The poor prognosis group was defined by detection of axillary lymph node metastasis, a tumor size with a diameter >2 cm, or negative results for ER or PR. Statistical analysis was done with SAS for Windows, version 9.0 (SAS Institute, Cary, NC, USA). Statistical significance was defined as a *P*- value <0.05.

For multivariate analysis of spectral data, Matlab (MathWorks, Natick, MA), SIMCA-P 11.0 (Umetrics,Sweden), and Excel (Microsoft, Seattle, WA) programs were used to process the numeric data. Principal component analysis, partial least square-discriminant analysis, and Orthogonal Projections to Latent Structure-Discriminant Analysis (OPLS-DA) were performed to distinguish patient groups by prognostic factors shown to have significant association with HR-MAS spectroscopic values in univariate analysis. Class discrimination models were built until the cross-validated predictability value did not meaningfully increase to avoid over-fitting of the statistical model. The statistical model was validated by prediction of unknown samples using a leave-one out analysis. An a priori cut-off value of 0.5 was used to evaluate the prediction results [22]. Signals contributing to class differentiation were identified by the S-plot and the corresponding metabolites were identified using Chenomx (Spectral database; Edmonton, Alberta, Canada) software and an in-house built database.

## Results

The mean tumor size was 29.7 mm (range, 10–80 mm). The most common tumor type was invasive ductal carcinoma (n = 30), and other cancers were 3 DCIS, 1 mucinous carcinoma, 1 tubular carcinoma, and 1 invasive micropapillary carcinoma.

HR-MAS MR spectroscopy quantified and discriminated various choline metabolites in all 36 breast cancer tissues obtained with CNB (Table 2, Fig. 1). The mean and median values of tCho concentration were 1.95 µmol/g (range, 0.06–6.55) and 1.46 µmol/g (interquartile range 0.61–3.0), respectively. The mean and median values of PC concentration were higher than those of Cho or GPC.

**Table 2:** HR-MAS MR spectroscopy values for 36 breast cancer specimens.

Metabolite concentration (µmol/g)		Metabolic ratio	
Metabolite	Mean±SD (Median)	Ratio	Mean±SD (Median)
Cho	0.36±0.28 (0.29)	Cho/Cr	1.76±0.99 (1.53)
PC	1.34±1.26 (0.88)	PC/Cr	1.77±1.20 (1.57)
GPC	0.25±0.24 (0.19)	GPC/Cr	1.06±0.75 (0.83)
tCho	1.95±1.63 (1.46)	tCho/Cr	4.60±2.17 (3.86)
Cr	0.66±0.47 (0.61)	GPC/PC	0.73±0.61 (0.60)
Gly	0.96±1.17 (0.62)	GPC/Cho	0.72±0.63 (0.55)
Tau	1.83±1.41 (1.56)	PC/Cho	1.28±1.08 (1.20)
s-Ins	0.56±0.58 (0.44)		
m-Ins	0.86±0.88 (0.53)		
Ala	0.79±0.69 (0.62)		
Suc	0.15±0.13 (0.09)		

Data represent the mean ± standard deviation (median).

Cho: choline, PC: phosphocholine, GPC: glycerophosphocholine, tCho: total choline (the sum of Cho, PC, and GPC), Cr: creatine, Tau: taurine, Gly: glycine, m-Ins: myo-inositol, s-Ins: scyllo-inositol, Ala: alanine, Suc: succinate.

As shown in Table 3, we found that several metabolite markers identified on HR-MAS MR spectroscopy that correlated with histopathologic prognostic markers. ER negative cancers showed higher Cho concentrations than ER positive cancers (p = 0.03). PR negative cancers showed higher concentrations of Cho, Cr, and Tau than those of PR positive cancers (p = 0.01, p = 0.02, and p = 0.02, respectively). Between HER2 positive and negative groups, concentrations of Tau, s-Ins, and m-Ins of HER2 positive cancers were significantly higher than those of the HER2 negative cancers (p = 0.01, p = 0.03, and p = 0.01, respectively). Cancers with high histologic grade showed higher PC/Cr than those of cancers with low histologic grade (p = 0.04). Triple negative cancers showed higher Cho concentrations and higher values of Cho/Cr and tCho/Cr than those of non-triple negative cancers. Cancers strongly positive for Ki-67 showed higher concentrations of tCho and PC, and higher values of PC/Cr than those of cancers weakly positive for Ki-67 (p = 0.01). The poor prognosis group showed higher Gly and s-Ins concentrations than those of the good prognosis group (p = 0.02 and p = 0.01, respectively). The histologic grade, tumor size, and lymph node metastasis did not correlate with HR-MAS MR spectroscopic values.

**Table 3:** Correlation between histopathologic parameters and HR-MAS MR spectroscopy values.

	ER			PR			HER2			Histologic Grade			Triple negative			Ki-67			Prognosis		
	Negative (n = 6)	Positive (n = 28)		Negative (n = 18)	Positive (n = 16)		Negative (n = 29)	Positive (n = 5)		Low (n = 26)	High (n = 10)		Negative (n = 29)	Positive (n = 4)		Low (n = 26)	High (n = 6)		Good (n = 9)	Poor (n = 25)	
Metabolite (concentration) or Metabolic ratio	Mean (SD)	Mean (SD)	*P*	Mean (SD)	Mean (SD)	*P*	Mean (SD)	Mean (SD)	*P*	Mean (SD)	Mean (SD)	*P*	Mean (SD)	Mean (SD)	*P*	Mean (SD)	Mean (SD)	*P*	Mean (SD)	Mean (SD)	*P*
Cho	**0.60 (0.17)**	**0.34 (0.28)**	**0.03**	**0.50 (0.28)**	**0.25 (0.23)**	**0.01**	0.37 (0.30)	0.47 (0.20)	0.50	0.34 (0.30)	0.40 (0.22)	0.58	**0.35 (0.27)**	**0.65 (0.20)**	**0.04**	0.35 (0.29)	0.49 (0.21)	0.28	0.24 (0.24)	0.43 (0.28)	0.07
PC	2.18 (1.78)	1.16 (1.05)	0.07	1.61 (1.31)	1.02 (1.03)	0.17	1.33 (1.38)	1.96 (0.63)	0.32	1.18 (1.15)	1.74 (1.52)	0.24	1.21 (1.03)	2.30 (2.29)	0.10	**1.08 (1.07)**	**2.60 (1.43)**	**0.01**	1.27 (1.27)	1.45 (1.29)	0.72
GPC	0.27 0.20)	0.25 (0.24)	0.80	0.30 (0.25)	0.19 (0.18)	0.15	0.23 (0.24)	0.39 (0.23)	0.19	0.24 (0.19)	0.25 (0.26)	0.89	0.25 (0.23)	0.26 (0.24)	0.93	0.23 (0.24)	0.37 (0.17)	0.20	0.24 (0.23)	0.27 (0.24)	0.76
tCho	3.06 (2.05)	1.75 (1.43)	0.07	2.42 (1.65)	1.47 (1.42)	0.08	1.94 (1.75)	2.83 (1.03)	0.28	1.78 (1.54)	2.39 (1.87)	0.32	1.81 (1.40)	3.21 (2.62)	0.10	**1.66 (1.47)**	**3.46 (1.71)**	**0.01**	1.75 (1.68)	2.16 (1.62)	0.53
Cr	0.82 (0.37)	0.64 (0.47)	0.39	**0.84 (0.47)**	**0.48 (0.36)**	**0.02**	0.60 (0.41)	1.04 (0.62)	0.06	0.66 (0.50)	0.68 (0.38)	0.88	0.86 (0.75)	0.59 (0.20)	0.74	0.62 (0.48)	0.94 (0.32)	0.14	0.51 (0.45)	0.77 (0.46)	0.15
Tau	2.60 (1.10)	1.71 (1.45)	0.17	**2.42 (1.48)**	**1.25 (1.06)**	**0.02**	**1.54 (0.95)**	**3.32 (2.11)**	**0.01**	1.69 (1.50)	2.17 (1.15)	0.38	1.87 (1.49)	2.07 (0.90)	0.79	1.79 (1.53)	2.34 (1.19)	0.43	1.27 (1.12)	2.17 (1.43)	0.12
s-Ins	0.55 (0.28)	0.63 (0.67)	0.78	0.59 (0.27)	0.64 (0.85)	0.85	**0.43 (0.30)**	**0.82 (0.27)**	**0.03**	0.59 (0.67)	0.48 (0.27)	0.66	0.66 (0.64)	0.39 (0.11)	0.41	0.63 (0.67)	0.58 (0.30)	0.86	**0.25 (0.18)**	**0.73 (0.64)**	**0.01**
m-Ins	1.22 (0.39)	0.83 (0.97)	0.35	1.17 (0.97)	0.58 (0.72)	0.06	**0.71 (0.65)**	**1.75 (1.48)**	**0.01**	0.86 (1.01)	0.85 (0.44)	0.97	0.87 (0.95)	1.12 (0.45)	0.62	0.86 (1.02)	1.05 (0.30)	0.66	0.54 (0.44)	1.03 (0.98)	0.06
Gly	1.18 (0.79)	0.97 (1.29)	0.72	1.14 (1.22)	0.86 (1.23)	0.52	0.78 (1.00)	1.99 (1.96)	0.24	1.01 (1.32)	0.83 (0.72)	0.69	1.04 (1.28)	0.83 (0.41)	0.75	1.03 (1.33)	0.99 (0.86)	0.93	**0.51 (0.30)**	**1.18 (1.34)**	**0.02**
Cho/Cr	2.53 (1.30)	1.68 (0.88)	0.06	2.01 (1.15)	1.62 (0.77)	0.27	1.99 (1.12)	1.46 (0.30)	0.31	1.74 (1.13)	1.81 (0.52)	0.86	**1.68 (0.85)**	**2.98 (1.41)**	**0.01**	1.77 (0.91)	1.60 (0.62)	0.65	1.71 (1.01)	1.83 (1.03)	0.76
PC/Cr	2.50 (2.12)	1.63 (0.96)	0.11	1.78 (1.46)	1.80 (0.94)	0.96	1.78 (1.35)	2.16 (0.95)	0.56	**1.51 (0.81)**	**2.41 (1.78)**	**0.04**	1.66 (0.91)	2.73 (2.68)	0.10	**1.51 (0.92)**	**2.97 (1.92)**	**0.01**	2.17 (0.95)	1.68 (1.30)	0.31
GPC/Cr	1.01 (0.70)	1.09 (0.79)	0.82	1.09 (0.88)	1.06 (0.64)	0.91	1.07 (0.81)	1.22 (0.76)	0.71	1.05 (0.76)	1.08 (0.77)	0.93	1.08 (0.77)	1.07 (0.88)	0.99	1.01 (0.78)	1.33 (0.87)	0.38	1.32 (0.64)	1.01 (0.79)	0.30
tCho/Cr	6.05 (3.12)	4.41 (1.91)	0.10	4.89 (2.60)	4.49 (1.70)	0.61	4.85 (2.43)	4.85 (1.85)	0.99	4.32 (1.90)	5.30 (2.72)	0.23	**4.42 (1.85)**	**6.80 (3.73)**	**0.04**	4.30 (1.85)	5.91 (3.22)	0.11	5.20 (1.52)	4.52 (2.38)	0.43

* Other NMR values (GPC/Cho, GPC/PC, PC/Cho) were not significantly different by histopathologic parameters.

**Bold** indicates statistical significance (*P*<0.05).

**Figure 2:**
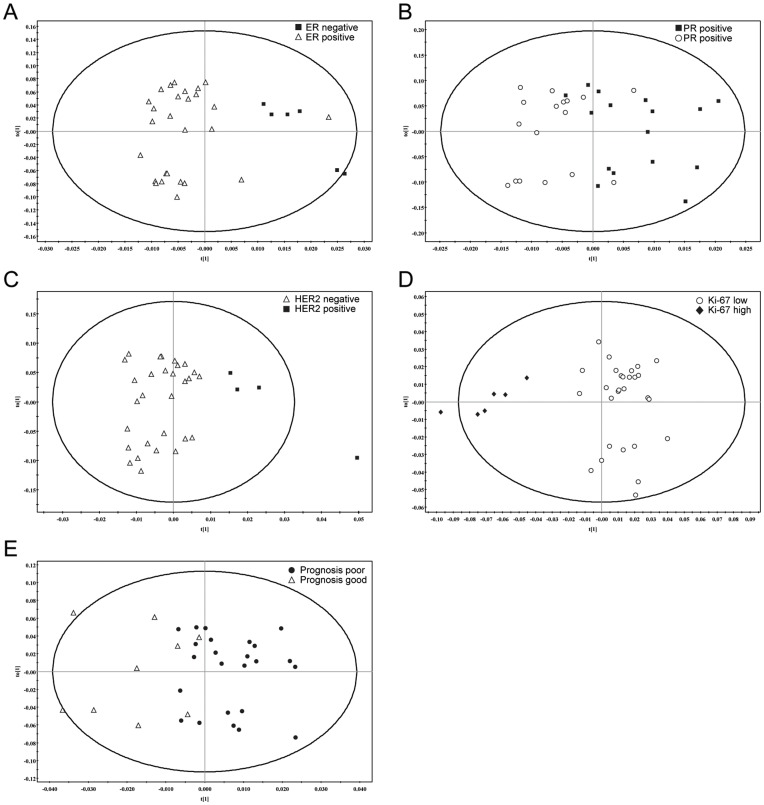
Orthogonal Projections to Latent Structure-Discriminant Analysis (OPLS-DA) score plot for estrogen receptor (ER), progesterone receptor (PR), HER2, Ki-67 status, and prognosis.

OPLS-DA separation models were produced with the HR-MAS MR spectral data by prognostic factors shown to have significant association in univariate analysis (ER, PR, HER2, Ki-67 status and prognosis). OPLS-DA score plots showed visible discrimination by status of ER, PR, HER2, and Ki-67 (Fig. 2), although some samples crossed over the reference line. In addition, reasonable distinction was observed between the good and poor prognosis groups. Triple negativity status could not be evaluated due to the small number of triple negative cancer samples in our study (n = 4).

## Discussion

In breast cancer research, most previous studies employing HR-MAS MR spectroscopy have used surgically obtained tissue specimens [8], [15], [16]. Therefore, their results have not been able to strongly influence the diagnosis of breast tumors or the preoperative decision making stage at which cancer treatment is planned. In this study, we conducted HR-MAS MR spectroscopy using 14-gauge CNB specimens. The tCho concentration (range, 0.06–6.55 µmol/g) acquired in our study with 11.7-T HR-MAS MR spectroscopy using CNB specimens was consistent with values reported in previous studies that used surgical specimens for HR-MAS MR spectroscopy [16]. US-guided CNB is the most frequently used procedure for diagnosis of suspicious breast lesions, therefore HR-MAS MR spectroscopic values for US-guided CNB specimens can be clinically applicable for diagnosis or characterization of breast cancers to decide treatment options such as neoadjuvant chemotherapy. In addition, CNB samples can be re-used for later histopathologic examinations after HR-MAS MR spectroscopy, because HR-MAS MR spectroscopy does little damage to the integrity of the tissue samples during examination [14], [19].

Choline-containing compounds are associated with cell signaling, lipid metabolism and cell membrane integrity [23], [24]. Many studies have reported that choline-containing compounds, especially PC, are elevated in breast cancer tissue compared to its normal counterparts [11], [15], [16], [25]–[28]. The higher concentrations of choline-containing compounds in breast cancer tissue may be a consequence of overexpression and changes in the levels of choline kinase activity in response to demands from the cell for increased phospholipid synthesis [29], [30]. In this study, HR-MAS MR spectroscopy quantified choline-containing compounds in all 36 cancer CNB samples of our patient group. HR-MAS MR spectra discriminated and quantified PC, GPC, and Cho, which contribute to the tCho peak signal. Concentrations of PC, GPC, and Cho were consistent with previously reported values, and PC was the main contributor of the Cho peak signal [15], [16]. A previous study by Li et al. showed that HR-MA MR spectroscopy on breast tissues obtained with CNB could clearly discriminate between cancer and non-cancer samples using metabolomics, although the data did not show the absolute concentrations for each Cho metabolite in the tissue samples [19]. Considering this with our own results, it is possible to infer that HR-MAS MR spectroscopy using CNB specimens may have a potential role in the diagnosis of breast cancer, although we did not evaluate non-cancer samples.

Some HR-MAS MR spectroscopic values (Cho, PC, Tau, s-Ins, m-Ins, Gly, Cho/Cr, tCho/Cr, PC/Cr) were found to be significantly correlated with known histopathologic prognostic factors (ER, PR, HER2, and Ki-67 status, histologic grade, triple negativity) and the poor prognosis group. In this study, concentrations and metabolic ratios of choline-containing compounds were elevated in ER-negative, PR-negative, HER2-positive, high histologic grade, strongly Ki-67 positive, and triple negative breast cancers. In addition, concentrations of Gly and s-Ins were significantly higher in the poor prognosis group than those of the good prognosis group. Our study showed that ER, PR, HER2, Ki-67 status, and the prognosis group correlated with HR-MAS MR spectroscopic values on CNB cancer samples through a OPLS-DA multivariate analysis. These results suggest that the aggressiveness and proliferative activity of breast cancer correlate with HR-MAS MR spectroscopic values for tissue samples obtained using CNB. Li et al. also showed relevant correlation of HR-MAS MR spectral data of CNB cancer samples with PR and lymph node metastasis status [19], although our study could not confirm the latter as lymph node metastasis did not show significant association with spectroscopic values in our univariate analysis. However, they were not able to evaluate ER and HER2 status due to the small number of patients in their cancer patient group. Looking at the results of other previous studies with our own results together, we can therefore expect HR-MAS MR spectroscopic values to be used as reliable markers for prognosis prediction in breast cancer patients. In order to confirm the relationship between HR-MAS MR spectroscopic values and prognosis, future studies about whether or not HR-MAS MR spectroscopic values are independent factors for breast cancer prognosis will be needed for large numbers of cancers.

Principal component analysis (PCA) and partial least square-discriminate analysis (PLS-DA) have been used in breast cancer metabolomics [14], [31]. OPLS-DA, which was used for our multivariate analysis, is an extension of the PLS-DA featuring an integrated orthogonal signal correction. The main benefit of OPLS-DA lies in the ability to separate the class-orthogonal variations that can obscure class discrimination. To date, the OPLS-DA has shown its utility in classifying data that has large intra-group variations such as the MR spectroscopic data of our study [32], [33]. In our study, OPLS-DA models provided visible discrimination of HR-MAS MR spectral data by prognostic factors.

Among the HR-MAS MR spectroscopic values significantly correlated with histologic prognostic factors in this study, PC and Cho are already well-known markers for breast cancer [15], [16], [26], [34], [35]. On the other hand, recent reports state that elevated levels of Tau and Gly can also be associated with breast cancer tissue [15], [16], [36]. Tau is an amino acid involved in many essential biological functions such as antioxidation, membrane stabilization, and cell shrinkage during apoptosis [37]–[40]. Cao et al. reported that the treatment response of breast cancer patients receiving neoadjuvant chemotherapy was best predicted by Tau among various choline metabolites obtained with HR-MAS MR spectroscopy [36]. In this study, non-survivor had lower Tau concentration and significantly lower Tau/Gly ratio compared to survivors at pre- and post-treatment. Meanwhile, Gly is an amino acid known as a precursor to proteins and the role of Gly in the development or progression of cancer is currently unknown. However, Gly has been found to contribute to characterization of breast cancer tissue in previous reports [16], [36], [41]. Sitter el al. reported that Gly concentrations were significantly higher in breast cancers larger than 2 cm compared to those of smaller cancers [16]. Cao et al. showed survivors had a significant decrease in Gly concentration after neoadjuvant chemotherapy, while it remained unchanged in non-survivors [36]. Jain et al. recently identified the correlation of Gly consumption and synthesis with rapid cancer cell proliferation, and a higher expression of the mitochondiral Gly biosynthesis pathway is associated with higher mortality in breast cancer patients [41]. Although the precise mechanisms of these findings are not known, our study also showed higher concentrations of Tau and Gly being associated with PR negative/HER2 positive cancers and the poor prognosis group, respectively. Accordingly, we suggest that Tau and Gly may be reliable markers in predicting treatment response or in suggesting poor prognosis during therapeutic monitoring of breast cancer patients. Besides these markers, Ala, Suc, and several polyunsaturated fatty acids were reported as biomarkers related to metabolism in cancer [34], [42], [43]. We could quantify these metabolites using HR-MAS MR data of our breast cancer tissues, although these values did not show significant correlation with prognostic factors. Therefore, further studies to assess the most reliable marker among the various HR-MAS MR spectroscopic values will be needed for breast cancer research.

In vivo proton MR spectroscopy has been studied extensively as an adjunctive technique for breast cancer imaging. This is a method that can provide metabolic information about tumors noninvasively, while HR-MAS MR spectroscopy requires an invasive procedure (e.g. surgical excision, fine needle aspiration biopsy, CNB) to acquire tissue samples for analysis. In some of the latest studies, tCho peak (signal/concentration) obtained with in vivo MR spectroscopy has shown good performances in differentiating between malignant and benign breast lesions [44], [45], and also showed correlation with some histologic prognostic factors [46]–[48]. In these studies, tCho was associated with high histologic/nuclear grade, triple negative, ER-negative cancers [46]–[48]. However, our results showed that tCho concentrations of CNB samples were correlated only with cancers strongly positive for Ki-67. This discrepancy may be resulted from tumor heterogeneity. Our study and the previous studies above mentioned included larger breast cancers. Heterogeneous character of larger breast cancers may bring about variation in choline metabolite concentrations of breast lesions. In the study by Bolan et al., single-voxel MR spectroscopic data acquired from different region of the same 3-cm breast cancer showed different tCho concnentrations [49]. Therefore, single-voxel location of MR spectroscopy and CNB specimen used for HR-MAS MR spectroscopy may not represent the metabolic composition of the large tumors. On the other hand, our study showed ER negative, PR negative, and triple negative cancers, which were found to be correlated with tCho in the previous studies using in vivo MR spectroscopy, were correlated with Cho concentration. Cho is a contributor of tCho peak signal obtained with in vivo MR spectroscopy and was found to be higher in tumors larger than 2 cm in the previous study [16]. However, in vivo MR spectroscopy cannot discriminate individual choline metabolites, which contribute to the tCho signal, because it uses a relatively low magnetic field strength (1.5–3.0 T) compared to HR-MAS MR spectroscopy. Considering these and our results, the correlation between tCho obtained with in vivo MR spectroscopy and the prognostic factors (ER and PR status, triple negativity) may result from higher Cho concentrations of the tumors. Therefore, comparative study on choline markers of in vivo MR spectroscopy and HR-MAS MR spectroscopy using CNB specimen may be needed to identify and verify correlation between them. And then, if there is the correlation between in vivo MR spectroscopy and HR-MAS MR spectroscopy using CNB specimen through the comparison study, HR-MAS MR spectroscopy using breast tissue obtained by minimally invasive CNB may prove to be a clinically useful approach to provide metabolic information on tumors with respect to invasiveness and data quality. Also, if in vivo MR spectroscopy can use a high magnetic field strength (7.0 T) in the future, the HR-MAS MR metabolic profile compiled with CNB samples will be the foundation for future studies of high-field results in vivo MR spectroscopy.

This study has several limitations. First, we excluded tumors smaller than 1 cm in diameter and included a relatively small number of tissue samples, which may have affected the results. Second, we included breast cancers of variable size, and 12 tumors (33.3%) were more than 3 cm in diameter. Larger breast cancers are known to have heterogeneous histologic features, which may induce variation in choline metabolite concentrations throughout the breast lesion. However, we did not compare HR-MAS MR spectroscopic values obtained with CNB specimens with the spectroscopic values of whole tumors (e.g. HR-MAS MR spectroscopic values obtained with surgical specimen, in vivo multivoxel MR spectroscopic values of whole tumor). Therefore, we cannot exclude the possibility that the CNB specimen used for HR-MAS MR spectroscopy may not fully represent the metabolic composition of the larger tumors. Finally, adjustments of acquisition parameters are important in obtaining optimal spectral data for HR-MAS MR spectroscopy. We tried to adjust the acquisition parameters in each case when undertaking HR-MAS MR spectroscopy in this study.

In conclusion, metabolic markers acquired with HR-MAS MR spectroscopy using CNB specimens showed significant correlation with histologic prognostic factors [ER, PR, HER2, triple negativity, histologic grade, Ki-67, and poor prognosis (detection of axillary lymph node metastasis, tumor size with a diameter >2 cm, or negative for ER or PR)]. Our results indicate HR-MAS MR spectroscopy using CNB specimens can be used to predict tumor aggressiveness prior to surgery in patients with breast cancer. In addition, it may be helpful for the detection of reliable markers of breast cancer characterization.
